# Ticks elicit variable fibrinogenolytic activities upon feeding on hosts with different immune backgrounds

**DOI:** 10.1038/srep44593

**Published:** 2017-03-16

**Authors:** Ashish Vora, Vikas Taank, Sucharita M. Dutta, John F. Anderson, Durland Fish, Daniel E. Sonenshine, John D. Catravas, Hameeda Sultana, Girish Neelakanta

**Affiliations:** 1 Department of Biological Sciences, Old Dominion University, Norfolk, VA, USA; 2 Leroy T. Canoles Jr. Cancer Research Center, Eastern Virginia Medical School, Norfolk, VA, USA; 3 Department of Entomology, Connecticut Agricultural Experiment Station, New Haven, CT, USA; 4 School of Public Health, Yale University School of Medicine, New Haven, CT, USA; 5 Frank Reidy Research Center for Bioelectrics, Old Dominion University, Norfolk, VA, USA.; 6 School of Medical Diagnostic and Translational Sciences, College of Health Sciences, Old Dominion University, Norfolk, VA, USA.; 7 Center for Molecular Medicine, College of Sciences, Old Dominion University, Norfolk, VA, USA

## Abstract

Ticks secrete several anti-hemostatic factors in their saliva to suppress the host innate and acquired immune defenses against infestations. Using *Ixodes scapularis* ticks and age-matched mice purchased from two independent commercial vendors with two different immune backgrounds as a model, we show that ticks fed on immunodeficient animals demonstrate decreased fibrinogenolytic activity in comparison to ticks fed on immunocompetent animals. Reduced levels of D-dimer (fibrin degradation product) were evident in ticks fed on immunodeficient animals in comparison to ticks fed on immunocompetent animals. Increased engorgement weights were noted for ticks fed on immunodeficient animals in comparison to ticks fed on immunocompetent animals. Furthermore, the LC-MS/MS and quantitative real-time-PCR analysis followed by inhibitor and antibody-blocking assays revealed that the arthropod HSP70-like molecule contributes to differential fibrinogenolysis during tick feeding. Collectively, these results not only indicate that ticks elicit variable fibrinogenolysis upon feeding on hosts with different immune backgrounds but also provide insights for the novel role of arthropod HSP70-like molecule in fibrinogenolysis during blood feeding.

Ticks are obligate hematophagous ectoparasites that can transmit several pathogens to humans and animals^1^,^2^,^3^. Understanding molecular interactions at the tick-host interface involve knowledge of the participation of host defense mechanisms against tick infestations and counter measures employed by ticks^4^. Acquired resistance by the host to tick infestations involves both humoral and cellular immunoregulatory pathways that impair tick feeding, egg production and viability^5^. On the other hand, ticks suppress host antibody production, complement activation and cytokine production from both antigen-presenting cells and T cell subsets^5^,^6^,^7^.

In the United States, *Ixodes scapularis* ticks transmit *Borrelia burgdorferi*, the causative agent of Lyme disease, *Anaplasma phagocytophilum*, the agent of Human anaplasmosis, *Babesia microti*, the agent of Human babesiosis, *Ehrlichia muris*-like agent (EMLA), the agent of Human ehrlichioses, Powassan virus, the agent of encephalitis and *B. miyamotoi*, the agent of relapsing fever^1^,^8^,^9^,^10^. In contrast to soft ticks that feed for short periods (1–3 hours), *I. scapularis* ticks feed on a host for more extended periods, up to 5 or 6 days for nymphs and even longer for adults^11^. To establish a successful feeding niche to commence blood feeding and engorge to completion, *I. scapularis* ticks secrete several pharmacologically active molecules in their saliva that include but not limited to anti-hemostatic, anti-inflammatory, immunosuppressive and immunomodulators targeting several host immune pathways^4^,^5^,^7^,^12^,^13^. Tick-borne pathogens also use some of these important classes of molecules present in tick saliva to infect a vertebrate host^14^,^15^,^16^,^17^,^18^,^19^.

Over the past few years, several studies have explored the importance of pathogen modulation of tick gene expression during tick-pathogen interactions^20^,^21^,^22^,^23^,^24^,^25^. However, the influence of different genetic or immune backgrounds of the vertebrate hosts on tick gene expression and blood feeding has not yet been fully evaluated. As *I. scapularis* ticks express a variety of molecules to counter host immune defense responses, including those noted previously, we used these ticks as a model to address this important question. Studies have reported significant variations in many basic hematological and coagulation parameters among many mouse strains^26^,^27^. In addition, a recent study has shown that T-cells participate in coupling coagulation with inflammation^28^. These studies provide strong rationale for the current study to test whether variable genetic or immune backgrounds of murine host influences tick feeding and gene expression. The findings presented in this study report that the host’s genetic background and/or immune status does influence specific tick gene expression that subsequently impact variable fibrinogenolysis during feeding.

## Results

### Tick engorgement weights are increased upon feeding on immunodeficient mice

**Figure 1:**
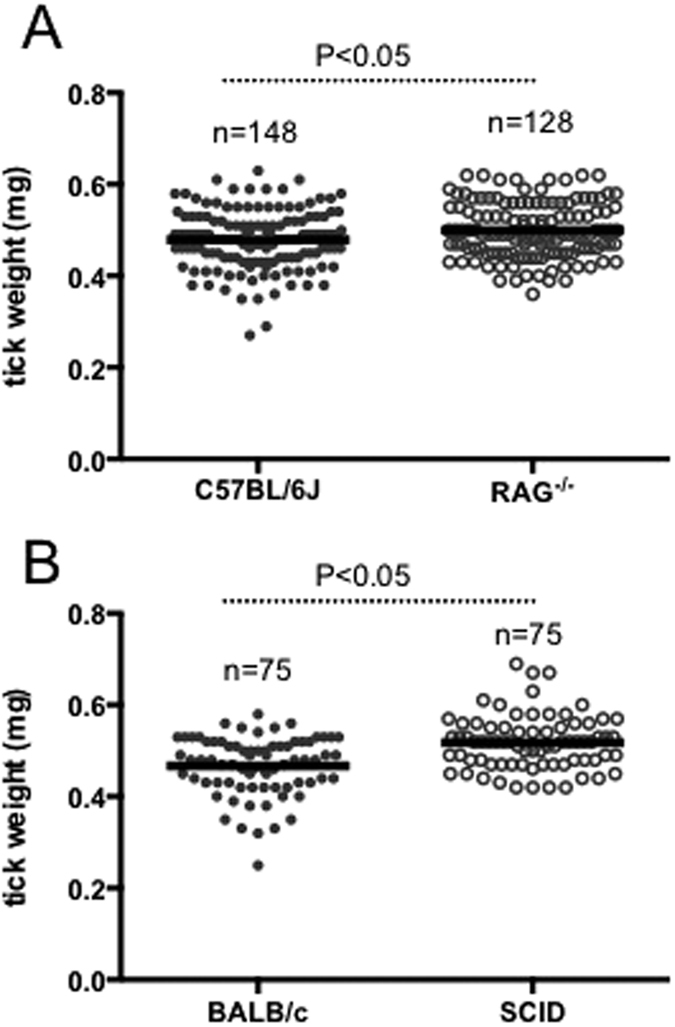
Engorgement weights of ticks are increased upon feeding on immunodeficient animals. Uninfected unfed larvae were fed on age- and gender-matched three immunocompetent mice (C57BL/6 J, BALB/c) or immunodeficient (RAG^−/−^, SCID) mice purchased from independent vendors. C57BL/6 J and RAG^−/−^ (**A**) mice are from Jackson laboratories and BALB/c and SCID NCr (**B**) mice are from Charles River Laboratories. Ticks were weighed soon after repletion. Engorgement weights are shown in milligrams and were measured using a Cahn C-31 microbalance set to a range from 25 mg to 0.1 μg. Student’s t test values are shown. Each circle represents one individual tick. Closed and open circles represent ticks fed on immunocompetent or immunodeficient mice, respectively.

We first analyzed whether the immune status of the animals influence tick feeding. Uninfected unfed larvae were fed on age and background matched immunocompetent (C57BL/6 J and BALB/c) or immunodeficient (RAG^−/−^ and SCID) mice ordered from two different commercial vendors with independent housing conditions (Jackson Laboratoreis-C57BL/6 J, RAG^−/−^ or Charles River Laboratories-BALB/c, SCID). Upon repletion, engorgement weights of fed larvae were measured using an analytical balance. We found that the engorgement weights of ticks fed on immunodeficient mice (0.499 ± 0.06 mg for RAG^−/−^; 0.517 ± 0.05 mg for SCID) were significantly (P < 0.05) higher in comparison to the ticks fed on immunocompetent (0.479 ± 0.06 mg for C57BL/6 J; 0.467 ± 0.06 mg for BALB/c) mice (Fig. 1A,B). The results were significant in both groups of mice ordered from two different vendors/housings (Fig. 1A,B). These results show significantly increased (P < 0.05) blood in-take by ticks upon feeding on immunodeficient animals in comparison to feeding on immunocompetent animals.

### Levels of host fibrin degradation product (D-dimer) are reduced in ticks acquiring blood from immunodeficient mice

**Figure 2:**
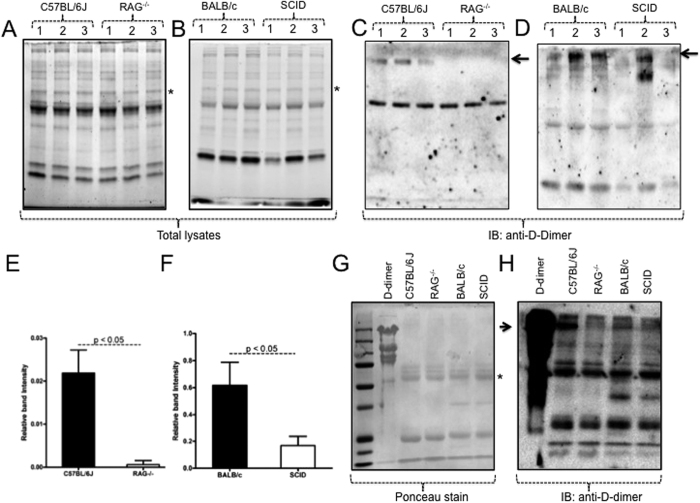
Levels of fibrin degradation product (D-dimer) are dramatically reduced in ticks fed on immunodeficient animals. Uninfected unfed larvae were fed on three (indicated as 1, 2, 3) immunocompetent (C57BL/6 J, BALB/c) or three immunodeficient (RAG^−/−^, SCID) mice. 3–5 ticks fed on each mouse were pooled and total lysates were generated separately. Total protein profile (**A**,**B**) from ticks fed on individual mouse (3 mice/group) is shown. Immunoblotting performed with 30 μg of tick total lysates and anti-D-dimer antibody showed dramatically low (**C**) or reduced (**D**) levels of D-dimer in ticks fed on immunodeficient animals in comparison to ticks fed on respective immunocompetent groups. Densitometry analysis showing levels of D-dimer observed in C57BL/6 J, RAG^−/−^ samples (**E**) and BALB/c, SCID samples (**F**) relative to the respective bands seen in A and B (marked with asterisk). Student’s t test values are shown. (**G**) Ponceau stained membrane image showing D-dimer (2 μg) protein (as control) along with tick total lysates (30 μg). Asterisk shows the position of the bands that were considered for densitometry analysis for relative quantification of D-dimer levels. (**H**) Immunoblotting assays with anti-D-dimer antibody showed presence of D-dimer in lysates prepared from ticks fed on immunocompetent animals (C57BL/6 J, BALB/c) at the same position (~200 kDa) as native D-dimer protein. Detection of lower bands in the D-dimer lane could be due to degradation of the native protein. Non-reducing and denaturation conditions were used in the SDS-PAGE analysis. 4–20% gradient SDS-PAGE gels (NuPAGE) gels were used for data in (**A–D**) and 10% laboratory-made SDS-PAGE gels were used for data in **G** and **H**. Immunoblotting with Anti-D-dimer antibody was performed at least three times. The Arrow next to the immunoblots (**C**,**D** and **H**) indicates position of D-dimer.

During feeding, ticks may acquire host proteins (including fibrin/fibrinogen or its degradation products) along with the vertebrate blood^29^. Therefore, we tested the levels of D-dimer, a prominent product of fibrin degradation^30^, in total lysates of whole ticks fed on immunodeficient or immunocompetent animals. Stain-free gel images showed no significant visual differences in the total protein profile between ticks fed on either group of mice (Fig. 2A,B). However, immunoblotting results showed dramatically low levels of D-dimer in the total lysates prepared from ticks fed on immunodeficient animals (RAG^−/−^, SCID) in comparison to the respective control group of ticks fed on immunocompetent (C57BL/6 J, BALB/c) animals (Fig. 2C,D, respectively). Densitometry analysis of the immunoblots further supported that these observations are statistically (P < 0.05) significant (Fig. 2E,F). An independent experiment further confirmed this observation (Supplementary Fig. 1A–C). Immunoblotting assays performed with commercially available D-dimer native protein (purified from human plasma, Lee Biosolutions) along with total lysates generated from ticks fed on immunocompetent (C57BL/6 J, BALB/c) animals further supported that the observed band at the size of ~200 kDa in these samples is indeed D-dimer which was absent in total lysates prepared from ticks fed on immunodeficient (RAG^−/−^, SCID) animals (Fig. 2G,H, Supplementary Fig. 2A,B). Densitometry analysis further supported the findings from the immunoblot analysis (Supplementary Fig. 2C). Collectively, these results provide evidence for a decreased level of blood clotting at the bite-site of ticks during feeding on immunodeficient animals.

### Salivary gland lysates prepared from ticks fed on immunodeficient animals show reduced fibrinogenolytic activity *in vitro*

**Figure 3:**
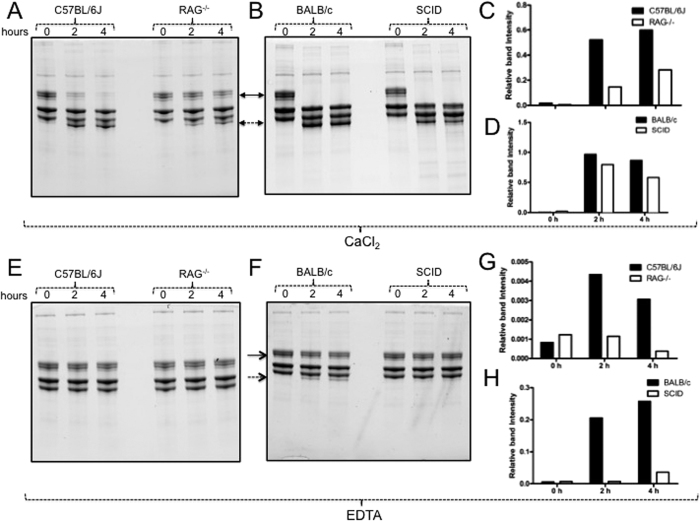
Salivary gland lysates prepared from ticks fed on immunodeficient animals show reduced *in vitro* fibrinogenolytic activity. Uninfected unfed nymphs were fed on immunocompetent mice (C57BL/6 J, BALB/c) or immunodeficient (RAG^−/−^, SCID) mice. Salivary glands were dissected from ticks fed on each group of mice, pooled and total lysates were generated separately. Fibrinogen (37.5 μg) was incubated with tick salivary gland lysates (5 μg) for the indicated times (in hours) in the presence of 1 mM CaCl_2_ (**A**,**B**,**C**,**D**) or 1 mM EDTA (**E**,**F**,**G**,**H**) in a total of 21 μl reaction volume. From the total volume, 3 μl was taken out at different time points, heated at 70 degrees with sample buffer to terminate the reactions and loaded on to a stain-free gel. 12% SDS-PAGE (BioRAD) gels were used for data in (**A**,**B**,**E**,**F**). Fibrinogenolysis assays were performed two times for samples prepared from ticks fed on C57BL/6 J and RAG^−/−^ mice and one time for samples generated from ticks fed on BALB/c and SCID mice. Solid arrow indicates Aα chain of fibrinogen and dotted line indicates degradation product. Densitometry analysis showing levels of degradation of Aα chain of fibrinogen in C57BL/6 J and RAG^−/−^ samples (**C**,**G**) or BALB/c and SCID samples (**D**,**H**) in the presence of CaCl_2_ (**C**,**D**) or EDTA (**G**,**H**) at the indicated time points for the images shown in (**A**,**B**,**C** and **D**). The levels of degradation product for each sample was measured relative to the respective levels of Aα chain at 0 min time point.

The reduced levels of D-dimer in ticks fed on immunodeficient animals suggest reduced fibrin clot formation at bite-site during blood feeding. Therefore, we analyzed *in vitro*, whether salivary gland lysates prepared from ticks fed on immunodeficient animals show reduced fibrinogenolytic activity in comparison to the lysates prepared from ticks fed on immunocompetent animals. Uninfected unfed nymphs were fed on immunocompetent (C57BL/6 J, BALB/c) or immunodeficient (RAG^−/−^, SCID) mice and upon repletion they were processed for salivary gland isolation as described^21^. Incubation of fibrinogen (purified protein from Sigma) with salivary gland lysates prepared from ticks fed on immunodeficient animals showed reduced degradation of Aα chain of fibrinogen in the presence of CaCl_2_ (when incubated for 2 or 4 h) in comparison to the lysates prepared from ticks fed on immunocompetent animals (Fig. 3A,B). Densitometry analysis further supported this observation (Fig. 3C,D). The salivary gland lysates prepared from ticks fed on immunocompetent (C57BL/6 J) mice showed increased degradation of Aα chain of fibrinogen in the presence of CaCl_2_ even at shorter incubation times (15, 30 min) in comparison to the lysates prepared from ticks fed on immunodeficient (RAG^−/−^) animals (Supplementary Fig. 3). The degradation of fibrinogen Aα chain (in all cases) was sensitive to EDTA treatment (Fig. 3E–H) in comparison to the CaCl_2_ treatment (Fig. 3A–D). Increased fibrinogenolytic activity was observed with the salivary gland lysates prepared from ticks fed on BALB/c mice even upon treatment with EDTA in comparison to the lysates prepared from ticks fed on SCID mice (Fig. 3F,H). Incubation of fibrinogen with CaCl_2_ or EDTA alone did not show any fibrinogen degradation (Supplementary Fig. 4). The amount of salivary gland lysates (5 μg) used in the fibrinogenolysis assay did not show any prominent band with the same intensity or size of the fibrinogen, ruling out the possibility of any tick molecules being detected at the degradation product size (Supplementary Fig. 4). These results indicate that variable fibrinogenolytic activities elicited by ticks are calcium dependent and sensitive to chelating agents such as EDTA.

### The gene expression for four metalloproteases analyzed in this study is unaltered in ticks fed on immunocompetent or immunodeficient animals

**Figure 4:**
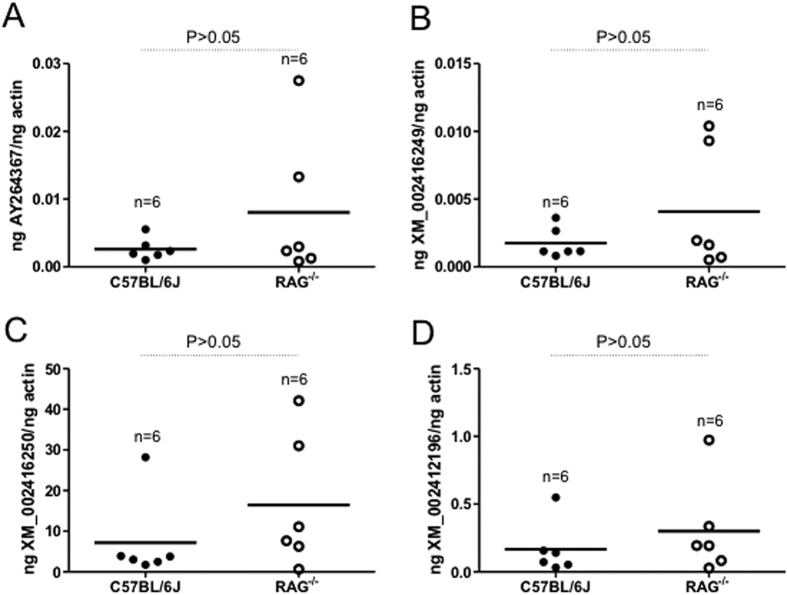
Expression of metalloproteases is unaltered in ticks upon feeding on immunocompetent or immunodeficient animals. (**A**) QRT-PCR results showing levels of four arthropod metalloproteases [GenBank acc. nos. AY264367 (**A**), XM_002416249 (**B**), XM–002416250 (**C**) and XM_002412196 (**D**)], transcripts in ticks fed on immunocompetent (C57BL/6 J, closed circles) or immunodeficient (RAG^−/−^, open circles) animals. The mRNA levels for metalloproteases were normalized to tick beta-actin. Each circle represents one tick. Student’s t test values are shown.

A tick metalloprotease belonging to reprolysin family containing pre- and pro-enzyme domains, zinc-binding motif and cysteine-rich region from salivary glands of *I. scapularis* has been suggested to participate in fibrinogenolytic activities^31^. Therefore, we analyzed expression of several of the tick metalloproteases (GenBank acc. nos. XM_002416249, XM_002416250 and XM_002412196) along with the salivary gland metalloprotease (GenBank acc. no. AY264367), that was previously suggested to participate in fibrinogenolysis^31^. QRT-PCR analysis revealed that expression of all four metalloproteases, analyzed in this study, did not show significant (P > 0.05) variable expression between ticks fed on immunocompetent (C57BL/6 J) or immunodeficient (RAG^−/−^) animals (Fig. 4A–D).

### Identification of proteins that show variable expression in salivary glands of ticks fed on immunocompetent versus immunodeficient animals

**Figure 5:**
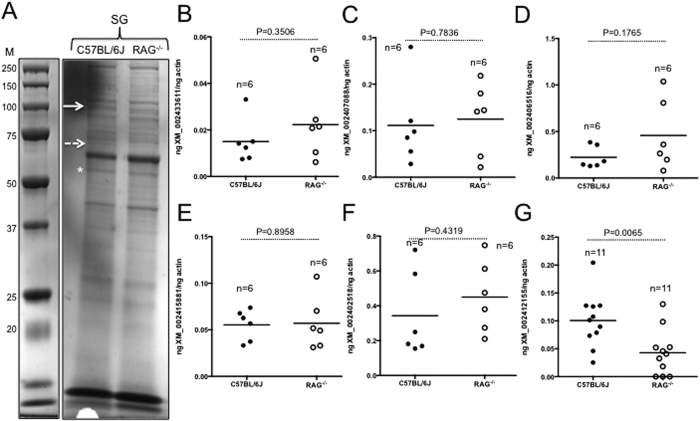
Transcripts of arthropod HSP70-like protein are down regulated in ticks upon feeding on immunodeficient animals. (**A**) 1-D SDS-PAGE (4–20% gradient gel, NuPAGE) analysis of total protein profile in salivary glands of ticks fed on immunocompetent (C57BL/6 J) or immunodeficient animals (RAG^−/−^) is shown. Solid arrow indicates band (that was excised and processed for LC-MS/MS analysis) around 100 kDa and dotted arrow indicates a band around 73 kDa that was found to be upregulated in salivary gland lysates prepared from ticks fed on immunocompetent mice. M indicates protein marker. Asterisk indicates position of control bands that were considered for densitometry analysis. SDS-PAGE analysis to study total protein profile in samples generated from ticks fed on C57BL/6 J or RAG^−/−^ animals was performed two times with different concentrations. QRT-PCR results showing levels of XM_002433611 (**B**), XM_002407088 (**C**), XM_002406516 (**D**), XM_002415881 (**E**), XM_002402518 (**F**) and XM_002412155 (**G**) transcripts in ticks fed on immunocompetent (C57BL/6 J, closed circles) or immunodeficient (RAG^−/−^, open circles) is shown. Levels of the transcripts for all *hsp70*-like molecules were normalized to tick beta-actin. HSP70 transcript XM_00241255 was undetectable or had very low threshold levels in some of the ticks that were fed on immunodeficient animals (RAG^−/−^). Therefore, additional tick samples were included in this assay. The amount of transcripts for *hsp70*-like molecules in tick samples that contained undetectable levels or well below threshold level were considered as “zero ng” for the analysis. Each circle represents one tick. Student’s t test P values are shown.

We then performed 1-D gel electrophoresis with the salivary gland lysates prepared from ticks fed on immunocompetent (C57BL/6 J) or immunodeficient (RAG^−/−^) animals (Fig. 5A). The results revealed increased band intensities for two of the bands at ~100 kDa and ~73 kDa band in lysates prepared from ticks fed on immunocompetent animals in comparison to the lysates prepared from ticks fed on immunodeficient animals (Fig. 5A and Supplementary Fig. 5A,B). Detection of ~100 kDa band was also evident in the salivary gland lysates prepared from ticks fed on BALB/c mice in comparison to lysates prepared from SCID mice (Supplementary Fig. 5C and D). Due to the detection of ~100 kDa band (with high intensity) in both groups of immunocompetent mice, this band from salivary gland lysates prepared from ticks fed on C57BL/6 J mice was excised and processed for LC-MS/MS analysis. The LC-MS/MS analysis on the ~100 kDa band revealed higher peptide matches for tick HSP70-like proteins (Supplementary Table 1, Supplementary Figs 6A,B).

### Level of one of the HSP70-like molecules is upregulated in ticks fed on immunocompetent animals in comparison to the immunodeficient animals

QRT-PCR analysis was performed with the samples generated from whole larval ticks fed on immunocompetent (C57BL/6 J) or immunodeficient (RAG^−/−^) mice for all six tick *hsp70*-like molecules that were identified in LC-MS/MS analysis (Supplementary Table 1). The mRNA levels for five *hsp70*-like molecules (GenBank Acc. Nos. XM_002433611, XM_002407088, XM_002406516, XM_002415881, and XM_002402518) were found to be unaltered in ticks fed on either group of mice (Fig. 5B–F). However, one of the HSP70-like molecules (GenBank Acc. No. XM_002412155) was noted to be significantly (P < 0.05) upregulated in ticks fed on immunocompetent mice in comparison to ticks fed on immunodeficient mice (Fig. 5G). Alignment of the amino acid sequences of all HSP70-like molecules analyzed in this study using Clustal W program revealed a high degree of conservation across the entire sequence (Supplementary Fig. 7). The tick HSP70-like molecule (XM_002412200) corresponding to the nucleotide sequence (GenBank acc. no. XM_002412155) shares approximately 82, 47, 72, 81, 59% identity with other tick HSP70-like molecules (GenBank acc. nos. XP-002406560, XP_002402562, XP_002407132, XP_002415926, XP_002433656) analyzed in this study (Supplementary Fig. 7). Taken together, the results from LC-MS/MS and QRT-PCR analysis provide important insights on the novel role for the participation of specific tick HSP70-like molecule in fibrinogenolysis during blood feeding.

### Treatment of salivary gland lysates prepared from ticks fed on immunocompetent animals with Ver155008 (HSP70 inhibitor) or with Anti-HSP70 antibody resulted in reduced fibrinogenolytic activity

**Figure 6:**
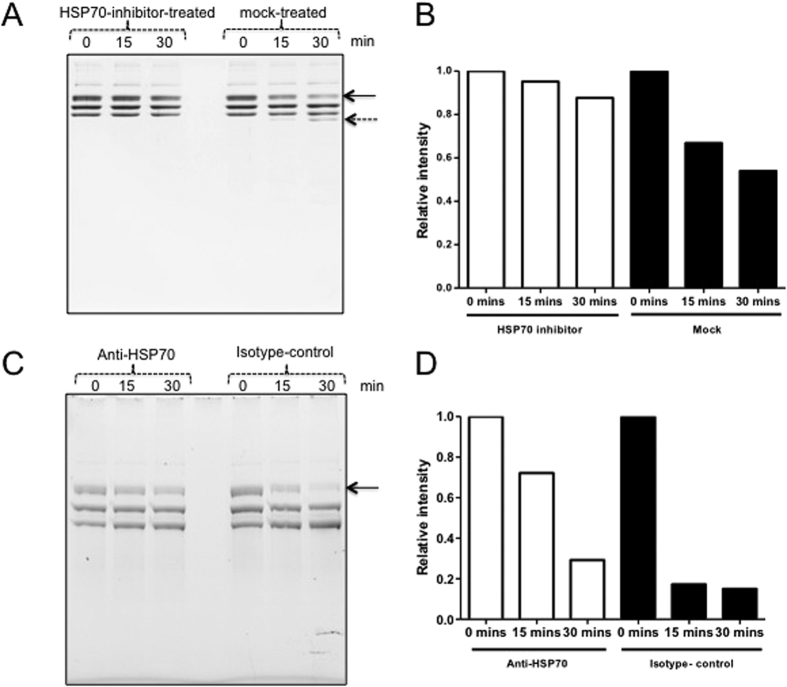
Tick HSP-70 like protein participates in variable fibrinogenolysis. Fibrinogenolysis assays performed with salivary gland lysates prepared from ticks fed on immunocompetent animals in the presence of 100 μM VER155008 (HSP70 inhibitor) or equal volume of mock control and assayed at indicated time points. 12% SDS-PAGE gel (Bio-Rad) was used in the assay. Fibrinogenolysis assays with VER155008 were performed at least three times. Solid arrow indicates increased Aα chain fibrinogen degradation and dotted arrow indicates increased level of fibrinogen-degraded product at 30 min time point in mock control in comparison to HSP70-inhibitor-treated samples. (**B**) Densitometry analysis (for image in (**A**) showing levels of degradation of Aα chain of fibrinogen in mock- or HSP70 inhibitor-treated samples at the indicated time points. (**C**) Fibrinogenolysis assays performed with salivary gland lysates prepared from ticks fed on immunocompetent animals in the presence of 50 ng of anti-HSP70 antibody or isotype control antibody and assayed at indicated time points is shown. Arrow indicates increased fibrinogen degradation in the presence of isotype-matched control antibody. 4–20% gradient SDS-PAGE gel (NuPAGE) gels were used in the analysis. Assays with Anti-HSP70 antibody was performed three times. (**D**) Densitometry analysis (for gel image in (**C**) showing levels of degradation of Aα chain of fibrinogen in isotype- or HSP70 antibody-treated samples at the indicated time points. In (**B** and **D**), the levels of Aα chain degradation for each sample was measured relative to the respective levels of Aα chain at 0 min time point.

To further validate whether HSP70-like molecules in ticks participate in fibrinogenolysis, salivary gland lysates prepared from ticks fed on immunocompetent animals (C57BL/6 J) were incubated with VER155008 (HSP70 inhibitor) or with equal volume of mock control for 90 min followed by incubation with fibrinogen (purified protein, Sigma) for 15 and 30 min. Salivary gland lysates treated with HSP70 inhibitor showed reduced Aα chain fibrinogenolysis in comparison to the mock-treated lysates (Fig. 6A,B). Independent assays also confirmed this observation (Supplementary Fig. 8A–D). Similar assays were performed with salivary gland lysates prepared from ticks fed on immunocompetent animals (C57BL/6 J) in the presence of anti-HSP70 antibody or isotype-matched control antibody (Fig. 6C). Salivary gland lysates treated with anti-HSP70 antibody showed reduced Aα chain fibrinogenolysis in comparison to the isotype-treated controls at 15 and 30 min incubation (Fig. 6C,D). Similar observation was evident at higher incubation time (45 min, 60 min and 2 h) points (Supplementary Fig. 9A–D). Collectively, these results support a novel role for the participation of tick HSP70-like molecule in variable fibrinogenolysis during blood feeding on hosts with different immune backgrounds.

## Discussion

To acquire a blood meal, ticks mechanically attach to their vertebrate host, insert their mouthparts in to the host skin, secrete saliva to encounter host defense mechanisms and cement themselves to the attachment site, engulf blood and then fall off upon completion of blood feeding^4^,^5^,^7^,^12^,^13^,^32^. Several tick molecules play essential roles in the blood feeding^4^,^5^,^6^,^7^,^13^,^33^,^34^. In this study, we report a novel role for the participation of tick HSP70-like molecule in fibrinogenolysis during blood feeding.

HSP70s are usually 70 kDa proteins. Tian *et al*., (2011) have shown that antibody generated against recombinant *Haemaphysalis longicornis* HSP70 recognized bands of approximately 100, 72 and 28 kDa in the egg lysates prepared from these ticks^35^. The variation in sizes suggests that tick HSP70-like molecules could undergo several posttranslational modifications to an active form. A recent study has shown that HSP70 forms antiparallel dimers that are stabilized by post-translational modifications to position clients for transfer to HSP90^36^. Therefore, the observation of an HSP70-like peptide sequence (with highest peptide match and scores) in the LC-MS/MS analysis (for a ~100 kDa band) in our study is not surprising.

Fibrinogen is an important factor in normal vertebrate host blood clotting^37^,^38^. Our hypothesis is that increased engorgement weights in ticks fed on immunodeficient mice is due to the reduced fibrin/fibrinogen clots in these mice. Our finding that ticks fed on immunodeficient animals express low level of HSP70-like protein is consistent with the reduced fibrinogen degradation around the tick bite site on these animals in comparison to the immunocompetent animals. The low levels of murine D-dimer in ticks fed on immunodeficient mice in comparison to ticks fed on immunocompetent mice strongly support this hypothesis (Fig. 2). Low levels of fibrin/fibrinogen clots could account for less clotting that may subsequently affect increased blood in-take by ticks fed on the immunodeficient animals (Fig. 1) and possibly increased transmission of pathogens. The later argument is in agreement with a study that has reported an increased rate of transmission of pathogen from ticks to immunodeficient animals in comparison to transmission of pathogens from ticks to the immunocompetent animals^39^.

HSP70 was initially thought to be an intracellular protein. However, work from Tytell and Hightower laboratories provide evidence that HSP70 could transit through the extracellular spaces^40^,^41^. HSP70 is also found in the human serum in substantial quantities suggesting its role as an extracellular or secreted protein^42^. In addition, a proteasome component Sec61p and HSP70 are reported to be involved in the degradation of fibrinogen in the mammalian cells^43^. Our data provides the evidence that suggests contribution of arthropod HSP70-like molecule in fibrinogenolysis during tick blood feeding. The observation of higher mRNA levels for only one of the HSP70-like molecule out of six molecules analyzed in this study does not rule out the possibility for the participation of other five HSP70-like molecules at different stages of tick blood feeding. In addition, role for other HSP70-like molecules in fibrinogenolysis cannot be ruled out in ticks during feeding on diverse vertebrate hosts such as deer or other animals. Future comparative studies on all tick HSP70-like molecules would reveal whether they have similar or redundant function during arthropod blood feeding. We hypothesize that HSP70-like molecules are secreted in tick saliva and may aid in proper folding of proteins that are involved in the degradation of fibrinogen at the arthropod bite site to facilitate blood feeding. However, the contribution of arthropod HSP70 molecules to fibrinogenolysis either directly or indirectly would not rule out other salivary activities by these molecules important during feeding.

HSP70s are molecular chaperones that consist of three domains: N-terminal ATPase domain, substrate binding domain and C-terminal domain^44^. The N-terminal ATPase domain hydrolyses ATP to ADP. The HSP70-substrate interactions are determined by the nucleotide status of the N-terminal domain. In the ATP-bound state, HSP70s have a high association and dissociation rates for substrate exchange. In contrast, in the ADP-bound state, HSP70s have low substrate exchange rates^44^. VER155008, an ATP-competitive inhibitor, arrests the N-terminal domain of HSP70 that subsequently affect substrate binding^45^. The observation of reduced fibrinogenolysis upon treatment of salivary glands lysates generated from ticks fed on immunocompetent animals with VER155008 suggests that N-terminal domain of arthropod HSP70 is critical for the recognition of substrate proteins that could be involved in fibrinogen degradation. Future studies on these aspects will reveal interesting findings on the role for arthropod HSP70-like molecules in fibrinogenolysis.

RAG^−/−^ mice do not produce any mature T or B-lymphocytes and this is described as a “non-leaky” immune deficiency animal model^46^. Whereas, some SCID mice appear to develop few clones of T and B cells^47^. The differences in the *in vitro* fibrinogenolysis observed between salivary gland lysates prepared from ticks fed on SCID in comparison to ticks fed on RAG^−/−^ mice could be due to the two different genetic backgrounds (BALB/c versus C57BL/6 J, respectively) or due to the leakiness in the generation of some clones of T and B cells in the SCID mice. T and B-cells play important roles in the host immune responses against tick bites^4^,^5^,^6^,^7^. The lack of T and B cells or other metabolites in the host blood could also account for increased blood in ticks fed on immunodeficient animals independent of the host blood clotting factors. Several studies have now characterized various tick salivary proteins involved in T and B cell inhibition^48^,^49^,^50^. It remains interesting to analyze whether tick HSP70-like proteins affect activation of host T and B cells and thereby facilitate arthropod blood feeding. The finding of a decreased level of fibrinogenolysis from lysates prepared from ticks fed on T and B-cell deficient background host provides important future avenues in understanding the role of these cells and their relevant signaling pathways in the formation of fibrin/fibrinogen clots around the feeding cavity formed by the arthropod bites.

In summary, using *I. scapularis* ticks as a model we provide evidence that ticks elicit variable fibrinogenolysis upon feeding on hosts with different genetic background and/or immune status. This study is not only important in understanding the molecular basis of the interactions at the tick-host interface but may also potentially lead for the development of anti-tick vaccines to interfere with the life cycle of this and perhaps other medically important vectors.

## Methods

### Ticks and mice

Laboratory-reared specimens of *I. scapularis* ticks were used throughout the study. Ticks used in this study were larvae and nymphs obtained from a continuously maintained tick colony at the Connecticut Agricultural Experiment Station (New Haven, CT) or at the Department of Epidemiology and Public Health, Yale University (New Haven, CT). All animal work in this study was carried out in strict accordance with the recommendations in the Guide for the Care and Use of Laboratory Animals of the National Institute of Health. The protocol (#16-017) approved by the Institutional Animal Care and Use Committee (Animal Welfare Assurance Number: A3172-01) was used in this study. Animal husbandry was provided in accordance with criteria approved by the Association for Assessment and Accreditation of Laboratory Animal Care Program at Old Dominion University. Acepromazine (Phoenix, St. Joseph, Missouri) tranquilizer was administered to the animals prior to handling to minimize anxiety and/or discomfort and all efforts were made to minimize suffering. To generate fed larvae or nymphs, unfed ticks were allowed to feed on naïve 6–8 weeks C57BL/6 J and B6.129S7-*Rag1*^*tm1Mom*^/J (RAG^−/−^) mice (from Jackson Laboratories) or BALB/c and SCID NCr (from Charles River Laboratories) to repletion and were collected immediately after drop-off for measurements of tick weights and RNA extractions. At least three mice per experimental group were used. For D-dimer immunoblotting assays, ticks that were collected from individual mouse were used in the analysis. For salivary gland lysate preparation, ticks were pooled from three mice and used for further analysis. Tick rearing was conducted in a Parameter Generation and Control incubator (Black Mountain, North Carolina) at 23 °C with 95% relative humidity and a 14/10 hour light/dark photoperiod regiment.

### Fibrinogenolysis Assays

The fibrinogen (2.5 mg/ml) purchased from Sigma (USA) was dissolved in solution with pH 7.4 containing 50 mM HEPES and 150 mM NaCl and distributed in two sets, one with 1 mM CaCl_2_ and the other with 1 mM EDTA. Five micrograms (1 μg/ul) of salivary gland extracts were pre-incubated with 1 μl of 150 mM NaCl. The samples were incubated for 90 min. The whole mix of salivary glands with 150 mM NaCl was then added to 15 μl (37.5 µg) of fibrinogen solutions making up a total volume of 21 μl. From 21 μl reaction mix, 3 μl was immediately taken out and considered as 0 min time point sample. Incubation was then continued and 3 μl samples were collected at different time points (15, 30, 120 and 240 minutes). The collected samples were stored on ice until further use. All samples were heated at 70 °C for 3 min before loading on to 4–20% gradient stain-free SDS-PAGE gels (Advansta, Bioexpress, USA). In some cases, salivary gland lysates and 150 mM NaCl solution was incubated with mock solution (4 μl of solution containing 1:1 of 1x phosphate buffered saline:DMSO, that corresponds to equal volume of HSP70-inhibitor solution) or 100 μM Ver155008 (HSP70-inhibitor dissolved in 1:1 ratio of 1x PBS:DMSO) or with 50 ng of IgG isotype control (5 mg/ml) or 50 ng of anti-HSP70 (20 μg/ml) antibodies (Abcam and Cell Signaling technologies, USA, respectively). 12% SDS-PAGE gels were used for HSP70-inhibitor or antibody-blocking fibrinogenolysis assays. Images for fibrinogen degradation were captured using Chemidoc MP imager (Bio-Rad, USA).

### RNA isolation and quantitative real-time PCR

Total RNA from fed larvae or salivary glands from nymphs was generated using the Aurum Total RNA mini kit (Bio-Rad, USA) following the manufacturer’s instructions. RNA was converted to cDNA using cDNA synthesis kit (BioRad, USA). Standard curve was prepared using 10-fold serial dilutions starting from 1 ng to 0.00001 ng of known quantities of *hsp70*-like molecules *or* actin fragments and QRT-PCR reactions were performed as described^51^. The generated cDNA was used as a template for quantifying transcripts of tick *hsp70*-like molecules. As an internal control and to normalize the amount of template, *I. scapularis* actin transcripts were quantified using oligonucleotides 5′ GGTATCGTGCTCGACTC 3′ and 5′ CAGGGCGACGTAGCAG 3′. QRT-PCR was performed using iQ-SYBR Green Supermix (Biorad, USA). Following are the oligonucleotide primers used in this study: XM_002433611, 5′ GAGTTTTCAAGAATGGGCGTGT 3′ and 5′ GTGAGGCTTGCTGTTCTTGTCC 3′; XM_002407088, 5′ CGGCTGATCGGTCGTCGT 3′ and 5′ CGCTGCGAGTCGTTGAAGT 3′; XM_002406516, 5′ GCGGAACACGGAGAACACAGT 3′ and 5′ GAACCTCTTCCGCTCTCCCT 3′; XM_002412155, 5′ GATGACCCCAAGATTCAGCAG 3′ and 5′ GCCTCGGCTGTTTCTTTCATCT 3′; XM_002415881, 5′ GAAAGAAACGGCGGAGGCT 3′ and 5′ CGTGGGCTCGTTGATGATG 3′; XM_002402518, 5′ GTGTAGCAGTGATGGAAGGGAAGA 3′ and 5′ CAGAGAGGGTGTTGGACGCA 3′. For metalloproteases screening following are the oligonucleotides used: AY264367, 5′ GGGACTCAGCCTGAAATTGTGGA 3′ and 5′ GATTTTGAGCATCCTCTCTCCAGT 3′; XM_002416249, 5′ CTGGTAGTGCAGACGGTTGACA 3′ and 5′ GGCGTCATGGTTGGAATCTTGT 3′; XM_002416250, 5′ GACATCTACCACGACTCAACTCACA 3′ and 5′ GTGGTCTGCCGTTTTCGTGT 3′; XM_002412196, 5′ CCGAATGAACCTGGTCAATCA 3′ and 5′ CTGCGTCCTGCTTTTTGTGTTGT 3′.

### Tick weight measurements

Fed ticks were collected immediately after repletion, cleaned with brushes to remove any mice hair or feces and weighed using a Cahn C-31 microbalance (Thermoelectric Co., PA, USA) set to a range from 25 mg to 0.1 μg. Total body weight measurements were obtained by directly placing each tick on a balance pan. Data in Fig. 1 shows the total tick body weights for at least 75 larvae/group.

### Immunoblotting and Densitometry analysis

Total lysates from 5–7 ticks fed on C57BL/6 J or RAG^−/−^ mice or BALB/c or SCID NCr animals were prepared in modified RIPA buffer (BioExpress, USA) supplemented with EDTA-free protease inhibitor cocktail (Sigma, USA). Protein concentrations were determined by BCA protein assay kit (Pierce/ThermoScientific, USA). Thirty micrograms of total lysates from each group were loaded onto a 12% non-reducing stain-free SDS-PAGE gels (BioRad or NuPAGE, USA) for D-dimer immunoblotting. In addition, D-dimer native protein that was purified from human plasma was purchased from Lee Biosolutions (MO, USA) and was used in immunoblotting assays performed on 10% SDS-PAGE gels. The percentage of SDS-PAGE gels used in each assay is mentioned in the appropriate figure legends. The protein gels were further processed for immunoblotting as described^21^. D-dimer antibody (Biorbyt, UK) and Rabbit polyclonal anti-IgG HRP-conjugated antibody (Santa Cruz Biotechnology Inc., USA) was used to detect D-dimer under non-reducing conditions. ECL reactions were performed using Advanced WesternBright ECL HRP substrate kit (Advansta, USA) and chemiluminescence reaction images were captured using Chemidoc MP imager (BioRad, USA). Densitometry analysis was performed using image lab software 4 by measuring intensity of D-dimer bands relative to the control band (equal loading control) from the stain-free image. For fibrinogenolysis assays, densitometry analysis was performed by measuring intensity of the degraded product band relative to the level of fibrinogen Aα at 0 min time point. For densitometry analysis of HSP70-antibody blocking or HSP70-inhibitor based fibrinogenolysis assays, intensity of fibrinogen Aα chain levels at different time points (15, 30, 45, 60, 120, 240 min) were measured relative to intensity level of Aα chain at 0 min time point. As Aα chain band at 0 min was considered as control to calculate relative intensity for bands at other time points, the relative intensity for Aα chain at 0 min was considered as “1” (intensity of A alpha chain at 0 min/intensity of alpha chain at 0 min).

### Salivary gland dissection

Salivary glands and gut tissues were dissected from individual freshly fed nymphal ticks in sterile 1x phosphate buffer saline. Tissues were then pooled in one vial and homogenized using Kontes homogenizer and disposable pestle (VWR, USA). Protein concentrations were determined by BCA protein assay kit (Pierce/ThermoScientific, USA). Five micrograms of salivary gland extracts were used for fibrinogenolysis assays.

### Mass spectrometry analysis

The tick samples were run on a 1-D gel for dual purposes of sample concentration and sample clean-up. This gel is stained with Coomassie blue and then de-stained and washed with a series of three washing buffers (50 mM ammonium bicarbonate, 50% acetonitrile and 80% acetonitrile). The bound proteins were reduced with 1 ml of 40 mM dithiothreitol for 25 minutes at 56 °C. The gels were rinsed with 1 ml of 50 mM ammonium bicarbonate buffer and the reduced proteins were alkylated with 1 ml of 50 mM Iodoacetamide for 30 minutes at 25 °C in the dark with constant mixing. The Iodoacetamide was discarded and the gel bound proteins were digested with 0.5 ml of trypsin (20 ng/μl; Promega, Madison, USA) in 50 mM ammonium bicarbonate buffer at 37 °C with constant mixing for 12 h. After digestion, the tryptic fraction was collected, and the gels were washed with 50 mM ammonium bicarbonate to collect any remaining tryptic peptides. The eluent containing the tryptic peptides were dried using a Speed-Vac apparatus (Thermo Fisher Scientific, San Jose, CA) and stored at 4 °C prior to mass spectrometric analysis.

### Peptide/Protein acquisition by ESI-MS/MS analysis

The dried samples were dissolved with 20 μl of 0.1% formic acid/water. 2 μl of each sample was analyzed by LC/ESI-MS/MS using a Q-Exactive (Thermo Fisher Scientific, USA) mass spectrometer with an Easy NanoLC-1000 system using data dependent acquisition with dynamic exclusion (DE = 1) settings. The data dependent acquisition settings used was a top 12 higher energy collision induced dissociation (HCD) for the Q-Exactive MS. The Q-Exactive mass spectrometer was used with capillary temperature, 250 °C; spray voltage, 1600 V; and S-lens voltage, 55%. The automatic gain control (AGC) target was 3e6 for Full MS scans and 2e5 for MS/MS scans. Resolving power for Q-Exactive was set at 70,000 for the full MS scan, and 17,500 for the MS/MS scan at m/z 200. LC/ESI-MS/MS analyses were conducted using a C18 column (75 μm × 150 mm). The mobile phases for the reverse phase chromatography were (A) 0.1% HCOOH/water and (B) 0.1% HCOOH in acetonitrile. A four-step, linear gradient was used for the LC separation (2% to 30% B in the first 47 minutes, followed by 80%B in the next 1 minute and holding at 80% B for 12 minutes).

### Peptide/protein identification from tick samples

The Sequest algorithm was used to identify peptides from the resulting MS/MS spectra by searching against the combined tick protein database extracted from Uniprot using the appropriate taxonomy using Proteome Discoverer (version 1.3, Thermo Scientific). Searching parameters for parent and fragment ion tolerances were set as 20 ppm and 30 mmu for the Q Exactive MS. Other parameters used were a fixed modification of carbamidomethylation –Cys, variable modifications of oxidation (Met). Trypsin was set as the protease with a maximum of 2 missed cleavages. Raw files and proteome discoverer search results for the target protein were input into the quantitative package; Pinpoint (version 1.1, Thermo Scientific, USA). The raw file was imported into the software that analyzed the data and determined the optimal peptide targets for the protein of interest. The software determines the retention time window and accurate m/z ratio for each targeted peptide and produced a mass list used directly in the acquisition method for scheduled MS/MS. Target m/z lists were generated automatically based on tryptic cleavage, specified modifications and retention time on the chromatogram obtained from the gel spots. Quantitation analysis was performed in an automated fashion using the software with a 5 ppm window for extracted ion chromatograms. The optimized acquisition method for the targeted peptides was run in triplicate to validate the quantitative differences observed in the samples from the discovery experiment. Label free differential analysis was performed to compare the four sets of tick samples after sample normalization.

### Statistics

The statistical significance of differences observed in data sets was analyzed using GraphPad Prism6 software and Microsoft Excel. For data to compare two means, the non-paired Student *t* test was performed. P values of <0.05 were considered significant in all tests. Wherever necessary, statistical test and P values are reported.

## Additional Information

**How to cite this article**: Vora, A. *et al*. Ticks elicit variable fibrinogenolytic activities upon feeding on hosts with different immune backgrounds. *Sci. Rep.* **7**, 44593; doi: 10.1038/srep44593 (2017).

**Publisher's note:** Springer Nature remains neutral with regard to jurisdictional claims in published maps and institutional affiliations.

## Supplementary Material

Supplementary Information
