# Supplementary material for: Ticks elicit variable fibrinogenolytic activities upon feeding on hosts with different immune backgrounds
Source: Sci Rep. 2017 Mar 16;7:44593. doi: 10.1038/srep44593 (PMC5353578; doi:10.1038/srep44593)
Supplement: Supplementary Information [file srep44593-s1.pdf]

Ticks elicit variable fibrinogenolytic activities upon feeding on hosts with different immune backgrounds

Ashish Vora [1], Vikas Taank [1], Sucharita M. Dutta [2], John F. Anderson [3], Durland Fish [4], Daniel E. Sonenshine [1], John D. Catravas [5, 6], Hameeda Sultana [1,7, #] and Girish Neelakanta [1, 7, #]

Supplemental data

Supplementary Figure legends

Supplementary Figure 1. Independent experiment showing presence of D-dimer in ticks fed on immunocompetent animals. A) 3-5 larval ticks fed on C57BL/6J (immunocompetent) or $RAG^{-/-}$ (immunodeficient) was pooled and total lysates were generated. Total protein profile in the samples on a 12% stain-free gel (Bio-Rad) is shown. B) Immunoblotting performed with 30 μg of tick total lysates and anti-D-dimer antibody showed dramatically low levels of D-dimer in ticks fed on immunodeficient animals in comparison to ticks fed on immunocompetent animals. C) Densitometry analysis for image in (B) is shown. D-dimer levels were calculated relative to the respective bands seen in (A) at position marked with asterisk.

Supplementary Figure 2. Levels of D-dimer are reduced in ticks fed on immunodeficient animals. Inverted image (A) or low intensity image (B) for data in Figure 2H is shown. Immunoblotting assays with anti-D-dimer antibody showed presence of D-dimer in lysates prepared from ticks fed on immunocompetent animals (C57BL/6J, BALB/c) at the same position

(~ 200 kDa) as native D-dimer protein. 10% laboratory made SDS-PAGE gels were used for data in A & B. Immunoblotting with D-dimer antibody was performed at least three times. The Arrow indicates position of D-dimer. C) Densitometry analysis for the immunoblot in B showing levels of D-dimer in ticks fed on immunocompetent (C57BL/6J, BALB/c) or immunodeficient ($RAG^{-/-}$, SCID) animals. The levels of D-dimer for each sample was measured relative to the control band indicated with an asterisk in Figure 2G.

Supplementary Figure 3. Salivary gland lysates prepared from ticks fed on immunocompetent animals show increased fibrinogenolytic activity even at early time points. Uninfected unfed nymphs were fed on immunocompetent mice (C57BL/6J) or immunodeficient ($RAG^{-/-}$) mice. Salivary glands were dissected from ticks fed on each group of mice, pooled and total lysates were generated separately. Fibrinogen (37.5 μg) was incubated with tick salivary gland lysates (5 μg) for the indicated times (in minutes) in the presence of 1mM $CaCl_2$ (A) or 1mM EDTA (B) in a total of 20 μl reaction volume. From the total volume, 3 μl was taken out at different time points heated at 70 degrees with sample buffer to terminate the reactions and loaded on to stain-free gel (12%, Bio-Rad). Fibrinogenolysis assays were performed two times for samples prepared from ticks fed on C57BL/6J and $RAG^{-/-}$ mice. Solid arrow indicates Aα chain of fibrinogen and dotted line indicates degradation product.

Supplementary Figure 4. Treatment of $CaCl_2$ or EDTA alone had no effect on Fibrinogen degradation. SDS-PAGE analysis of salivary gland lysates (5 μg) prepared from ticks fed on immunocompetent (C57BL/6J, BALB/c) or immunodeficient animals ($RAG^{-/-}$, SCID) showed no prominent bands at the fibrinogen degradation product size. Incubation of fibrinogen with $CaCl_2$

or EDTA alone had no effect on fibrinogen degradation. 4-20% SDS-PAGE gradient gels (NuPAGE) were used in this assay. SDS-PAGE analysis with salivary gland lysates was performed two times with different concentrations.

Supplementary Figure 5. SDS-PAGE analysis of total protein profiles in ticks fed on immunocompetent or immunodeficient animals. Densitometry analysis for the SDS-PAGE gel image from Figure 5A showing levels of ~100 kDa (A) and ~ 73 kDa (B) protein band in ticks fed on immunocompetent (C57BL/6J) or immunodeficient ($RAG^{-/-}$) animals. The ~100 kDa protein band was analyzed by LC-MS/MS analysis. C) 1-D SDS-PAGE (10% laboratory-made gel) analysis of total protein profile in salivary glands of ticks fed on immunocompetent (BALB/c) or immunodeficient animals (SCID) is shown. Solid arrow indicates band around ~ 100 kDa that was found to be upregulated in salivary gland lysates prepared from ticks fed on BALB/c mice in comparison to samples generated from ticks fed on SCID mice. M indicates protein marker. Asterisk indicates position of control bands that were considered for densitometry analysis in (D).

Supplementary Figure 6. Extracted Ion Chromatogram from LC-MS/MS analysis. A) The Uniprot sequence for XM_002412155 identified in the MS analysis are colored in blue or red text. Orange triangles indicate oxidation sites and blue triangles indicate carbamidomethylation sites. B) The extracted Ion Chromatograms for all identified peptides for Uniprot sequence (B7Q7S0) with GenBank acc. no. XM_002412155 are shown.

Supplementary Figure 7. Alignment of *I. scapularis* HSP70-like molecules identified from LC-MS/MS analysis. Amino acid sequence alignment using Clustal W program in DNASTAR Lasergene is shown. Residues that match are shaded as black color. The GenBank accession numbers for proteins XP_002412200, XP_002406560, XP_002402562, XP_002407132, XP_002415926 and XP_002433656 corresponds to the nucleotide accession numbers XM_002412155, XM_002406516, XM_002402518, XM_002407088, XM_002415881 and XM_002433611, respectively. Percent identities for XP_002412200 (GenBank nucleotide acc. no. XM_002412155) in comparison to other HSP70-like molecules are shown at the bottom of the alignment.

Supplementary Figure 8: Inhibition of tick HSP-70 like proteins with VER155008 reduces fibrinogenolysis. A) Independent fibrinogenolysis assays performed with salivary gland lysates prepared from ticks fed on immunocompetent animals in the presence of 100 μM VER155008 (HSP70 inhibitor) or equal volume of mock control and assayed at indicated time points. 10% SDS-PAGE (laboratory made) gel was used in the assay. Fibrinogenolysis assays with VER155008 were performed at least three times. The gel is shown as an inverted image. Arrow indicates inhibition of Aα chain fibrinogen degradation at 30 min time point in the presence of HSP70 inhibitor in comparison to mock-treated control. B**)** Densitometry analysis (for image A) showing levels of degradation of Aα chain of fibrinogen in mock- or HSP70 inhibitor-treated samples at the indicated time points. C) Another independent round of fibrinogenolysis assays performed with salivary gland lysates prepared from ticks fed on immunocompetent animals in the presence of 100 μM VER155008 (HSP70 inhibitor) or equal volume of mock control and assayed at indicated time points. Arrow indicates reduced fibrinogen degradation in the presence

of HSP70 inhibitor. 4-20% gradient SDS-PAGE gels (NuPAGE) gels were used in the analysis. D) Densitometry analysis for gel image in Figure C is shown. The levels of Aα chain degradation for each sample was measured relative to the respective levels of Aα chain at 0 min time point.

Supplementary Figure 9: Inhibition of tick HSP-70 like proteins with anti-HSP70 antibody reduces fibrinogenolysis even at late time points. Fibrinogenolysis assays performed with salivary gland lysates prepared from ticks fed on immunocompetent animals in the presence of 50 ng of anti-HSP70 antibody or isotype control antibody and assayed at indicated longer time points: 45 and 60 min (A) or 2 and 4 h (B). The gel in A is shown as an inverted image. Arrows in A and B indicates reduced degradation of Aα chain of fibrinogen at 45 min, 60 min and 2 h time points, respectively in the presence of anti-HSP70 antibody in comparison to the isotype-control treated samples. Densitometry analysis for the image in A & B showing levels of degradation of Aα chain of fibrinogen in isotype- or HSP70 antibody-treated samples at the indicated time points: 45 and 60 min (C) or 2 and 4 h (D). 10% SDS-PAGE (laboratory-made) or 4-20% gradient gel was used for data in A and B, respectively. Fibrinogenolysis assays with Anti-HSP70 antibody was performed at least three times. The levels of degradation for each sample was measured relative to the respective levels of Aα chain at 0 min time point.

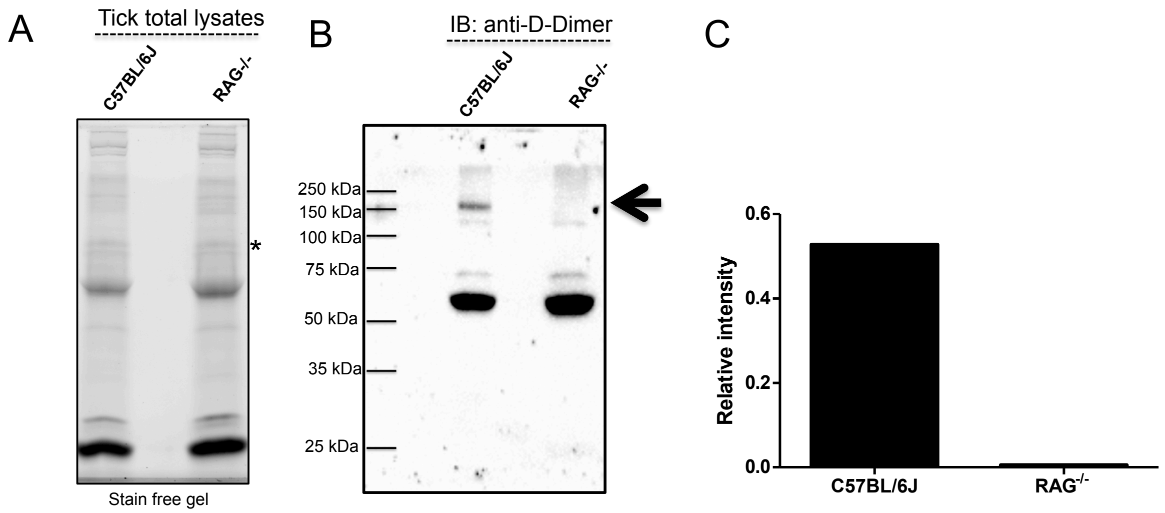

Supplementary Figure 1

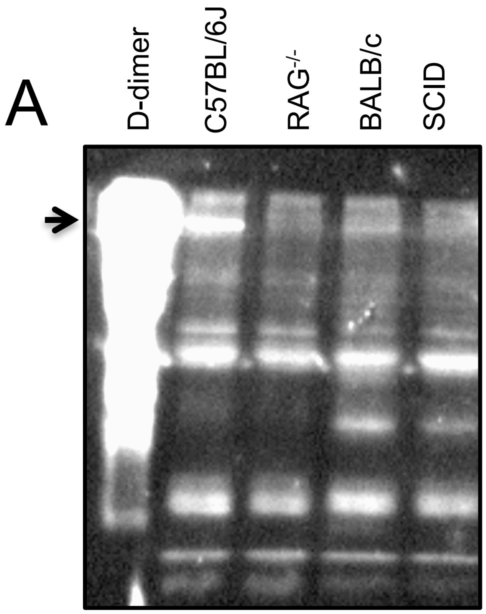

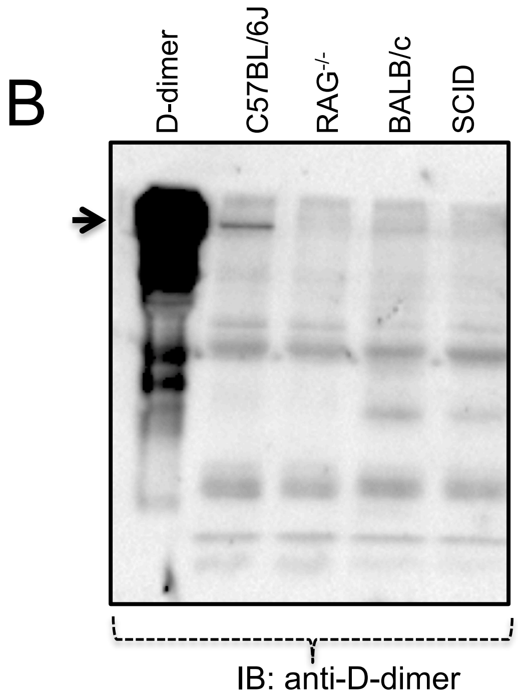

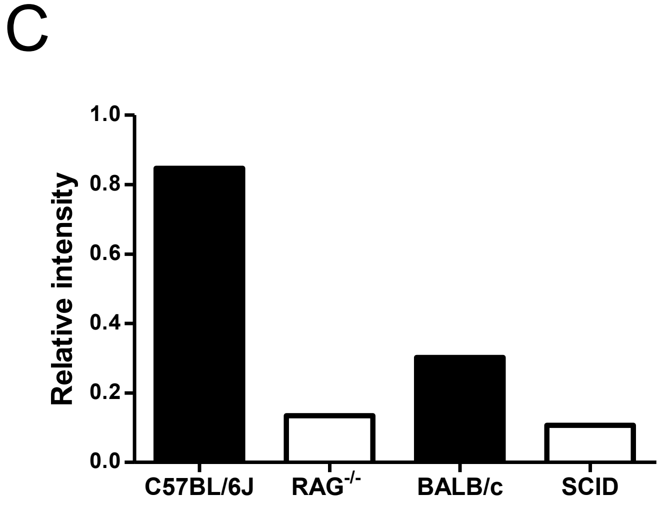

Supplementary Figure 2

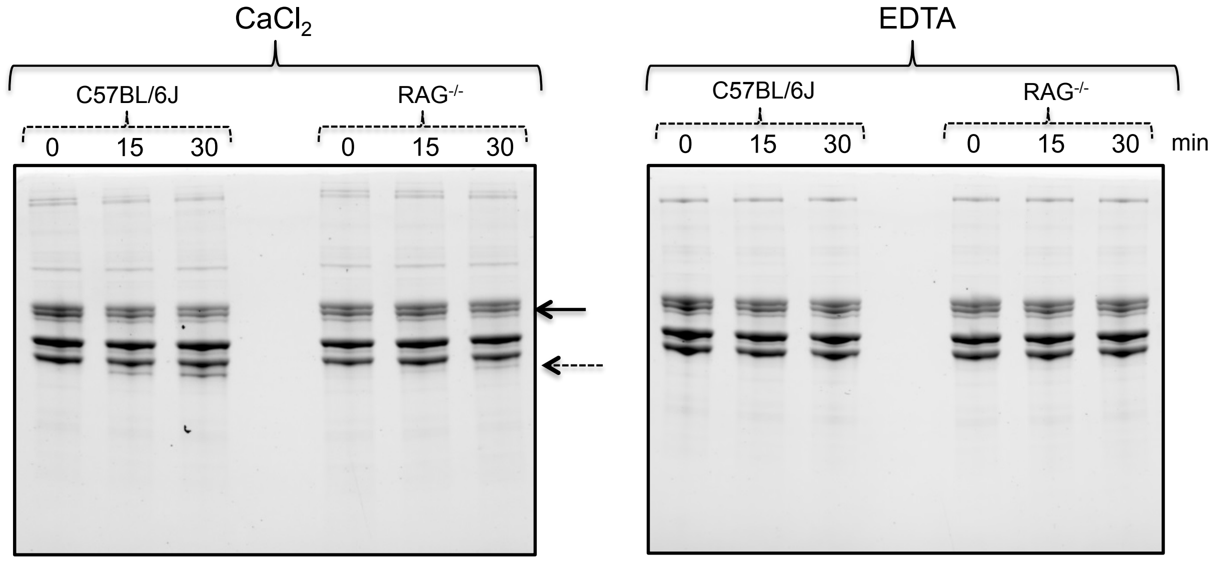

Supplementary Figure 3

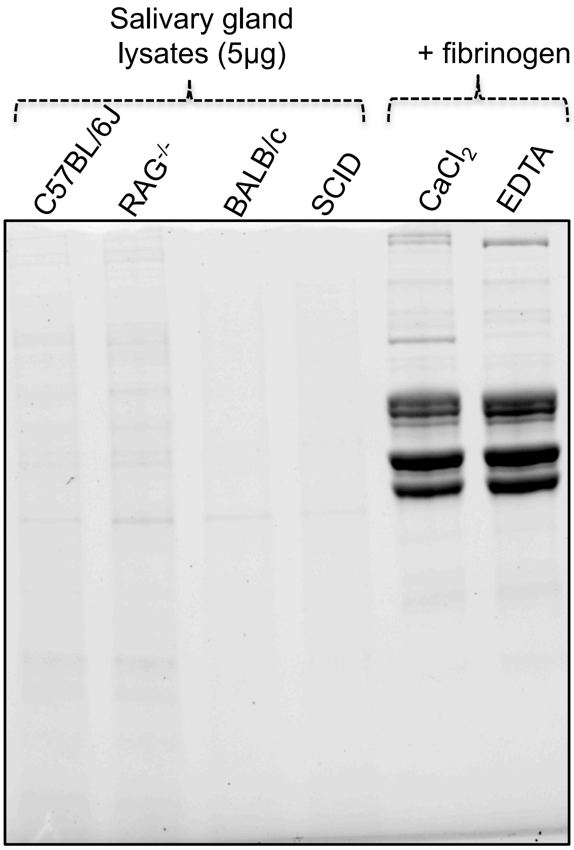

Supplementary Figure 4

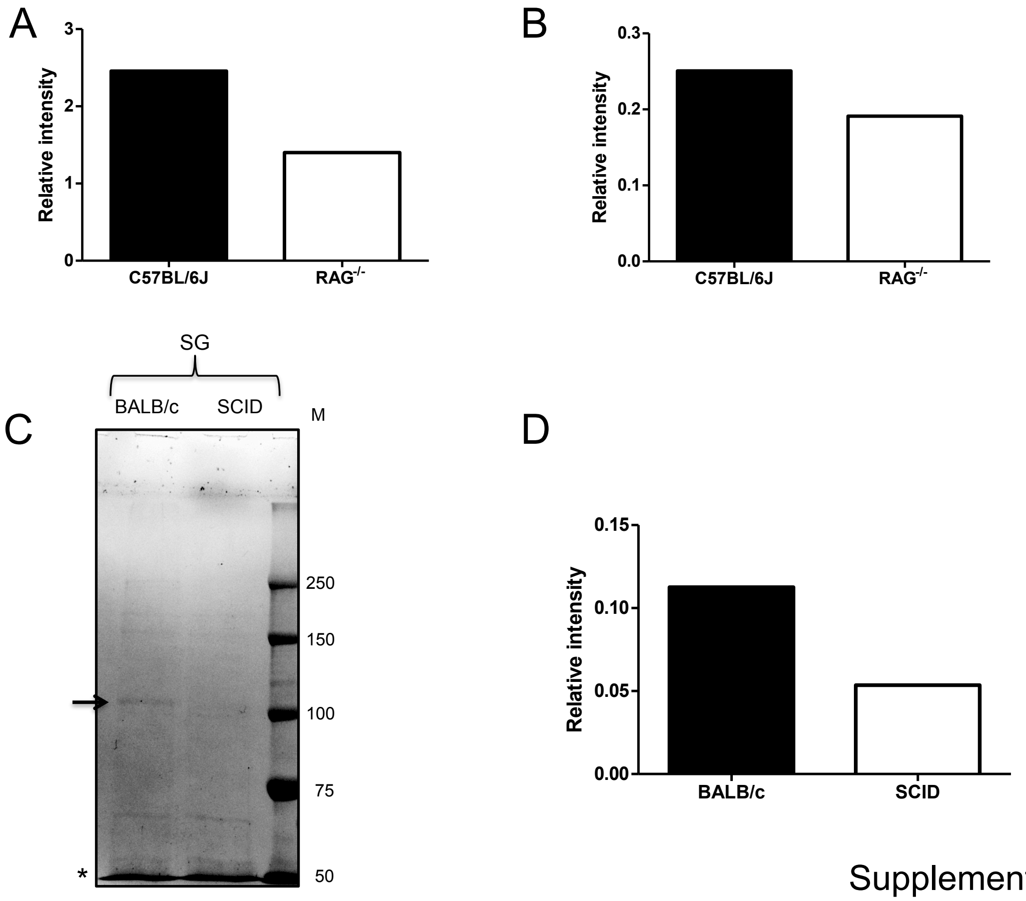

Supplementary Figure 5

A B

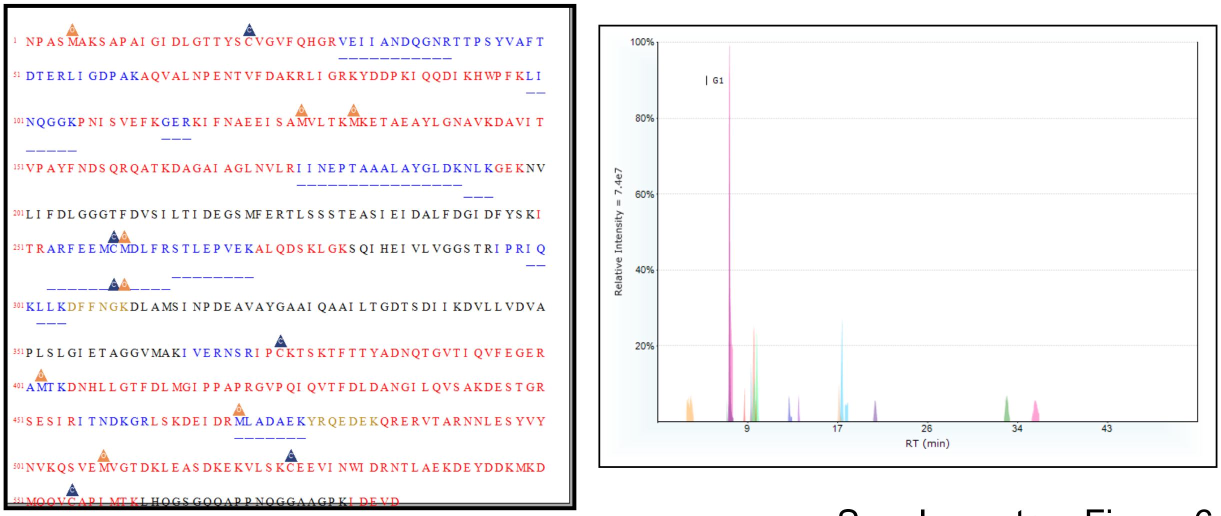

Supplementary Figure 6

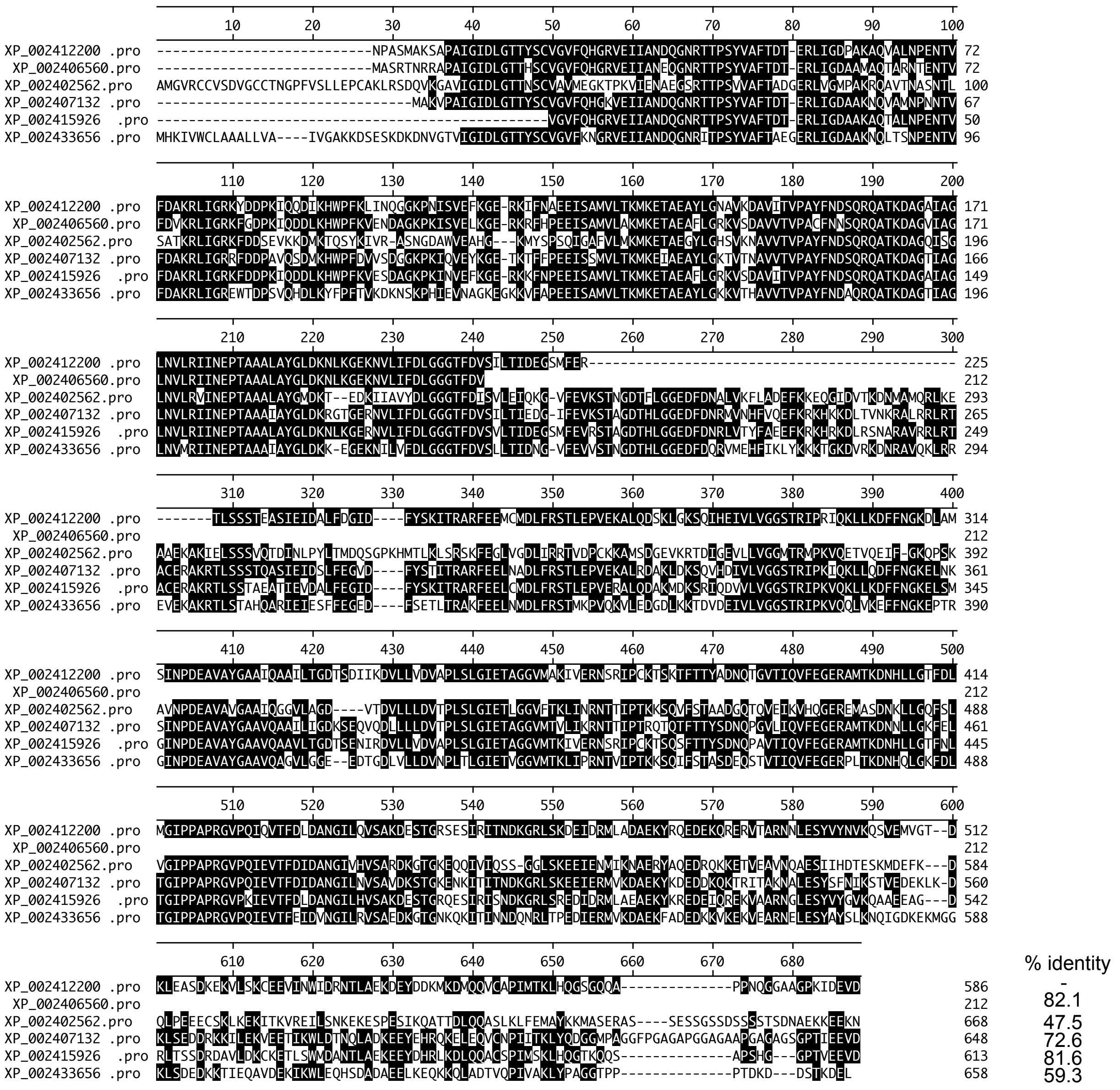

Supplementary Figure 7

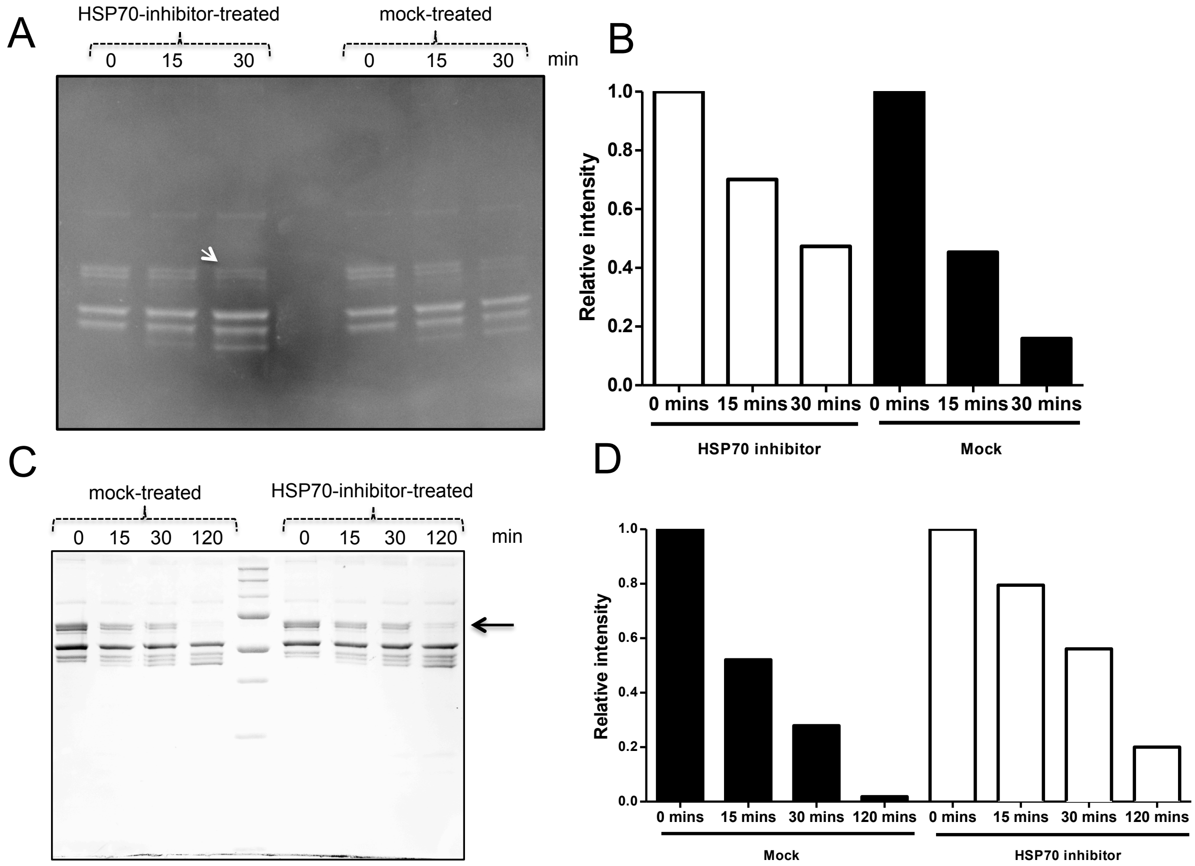

Supplementary Figure 8

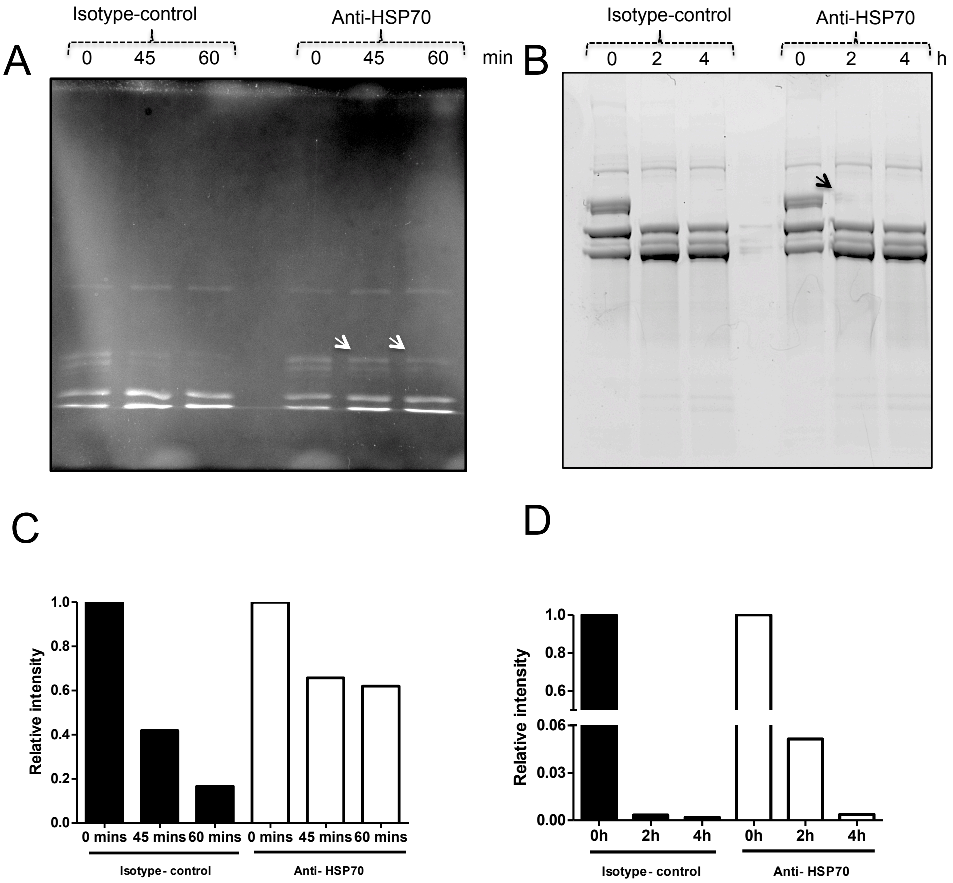

Supplementary Figure 9

Supplementary Table 1. LC-MS/MS data showing presence of arthropod HSP70-like proteins in salivary gland lysates prepared from ticks fed on immunocompetent mice

Uniprot accession number	Description	GenBank Accession number	# PSMs	Molecular Function	Biological Process	MW (kDa)
B7PEN4	Heat shock protein, putative OS=Ixodes scapularis GN=IscW_ISCW017754 PE=3 SV=1 - [B7PEN4_IXOSC]	XM_002433611	35	catalytic activity; nucleotide binding	cell organization and biogenesis; metabolic process; response to stimulus	72.5
B7PAR6	Heat shock protein, putative OS=Ixodes scapularis GN=IscW_ISCW017456 PE=3 SV=1 - [B7PAR6_IXOSC]	XM_002407088	41	catalytic activity; nucleotide binding	cell organization and biogenesis; metabolic process; response to stimulus	71.1
B7P4X5	Heat shock protein 70, putative (Fragment) OS=Ixodes scapularis GN=IscW_ISCW024057 PE=3 SV=1 - [B7P4X5_IXOSC]	XM_002406516	12	nucleotide binding	cell organization and biogenesis; response to stimulus	23.1
B7Q7S0	Heat shock protein, putative (Fragment) OS=Ixodes scapularis GN=IscW_ISCW011425 PE=3 SV=1 - [B7Q7S0_IXOSC]	XM_002412155	15	catalytic activity; nucleotide binding	cell organization and biogenesis; response to stimulus	64.3
B7QL71	Heat shock protein, putative (Fragment) OS=Ixodes scapularis GN=IscW_ISCW024910 PE=3 SV=1 - [B7QL71_IXOSC]	XM_002415881	14	nucleotide binding	cell organization and biogenesis; response to stimulus	67.8
B7P8Q5	Hsp70, putative (Fragment) OS=Ixodes scapularis GN=IscW_ISCW017192 PE=3 SV=1 - [B7P8Q5_IXOSC]	XM_002402518	14	catalytic activity; nucleotide binding; protein binding	cell organization and biogenesis; metabolic process	72.2
